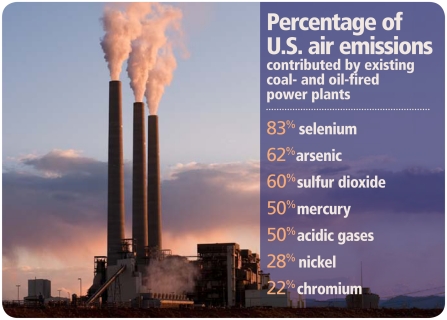# AIR POLLUTION: New Rules Proposed for Power Plant Toxics

**DOI:** 10.1289/ehp.119-a245

**Published:** 2011-06

**Authors:** Bob Weinhold

**Affiliations:** **Bob Weinhold**, MA, has covered environmental health issues for numerous outlets since 1996. He is a member of the Society of Environmental Journalists

Coal- and oil-fired power plants are key cogs in U.S. economic development. They’re also major emitters of many toxic substances, including mercury, arsenic, chromium, and dioxins. In an effort to dramatically cut the latter while modestly impacting the former, the U.S. Environmental Protection Agency (EPA) is proposing regulations that would set the first national standards for emissions of toxic substances from about 525 power plants.^1^,^2^ The agency estimates that by 2016 the proposed standards, which would regulate 67 toxics, could produce health benefits worth $5–13 (for avoided premature deaths, nonfatal heart attacks, respiratory problems, lost work days, and other health outcomes) for every $1 spent to meet the requirements.^1^ But Melissa McHenry, spokeswoman for American Electric Power, whose 25 coal-fired power plants serve 5.3 million customers in 11 U.S. states, says the agency is significantly underestimating costs to industry.

The new regulations, which are scheduled by consent decree to be finalized by 16 November 2011, would replace a George W. Bush administration regulation that addressed only mercury.^3^ In February 2008 the U.S. Court of Appeals for the District of Columbia vacated that regulation, deeming it inadequate under the requirements of the Clean Air Act.^4^

Among the toxics covered in the proposed rule for existing plants are mercury, lead, arsenic, chromium, cadmium, nickel, antimony, beryllium, manganese, hydrogen chloride (HCl), hydrogen fluoride (HF), dioxins, and furans. Additional sulfur dioxide (SO_2_), nitrogen oxides (NO_x_), and particulate matter (PM) standards would be implemented for new plants. The agency says existing plants, which are located in nearly every state and provide 46% of U.S. electricity generation, are responsible for 83% of all airborne selenium emissions, 62% of arsenic, 60% of SO_2_, 50% of mercury, over 50% of many acid gases (including HCl and HF), 28% of nickel, and 22% of chromium.^1^,^5^

The agency says existing technology^6^ could be used to meet all the proposed standards and that the installed equipment would concurrently reduce SO_2_, NO_x_, and PM even in existing plants for which such controls would not be mandated. As part of the new rules, the agency is also proposing mandatory work practices that would lead to optimal combustion and subsequent reductions in toxics such as dioxins and furans.

When installed, the EPA estimates the new equipment and operating practices would keep 91% of the mercury in coal from being released into the air and reduce 91% of acid gases and 55% of SO_2_ from power plants each year. The agency also predicts that implementing the controls will not only prevent 850,000 days of missed work each year but also provide 31,000 short-term construction jobs and 9,000 long-term utility jobs.^7^

Regarding the toxics parameters, McHenry says, “We don’t have a problem with the proposed limits.” But she is concerned about the time allotted by the EPA to implement necessary controls. Power plant owners and operators would have three years to comply after the regulations are finalized, with the possibility of an additional year in certain circumstances. That’s too tight, she says, especially if about 20% of all plants have to shut down rather than add emission controls, which is what she says the industry is estimating. She says those closures might make it difficult for the remaining plants to meet peak demands.

A 15 April 2011 press release issued by Southern Company, which has 4.4 million customers in four Southern states, quotes company head Thomas A. Fanning as saying, “As the CEO of a company that has installed more pollution controls than any other utility, I tell you that this cannot be done in three years.”^8^ Furthermore, Southern Company spokeswoman Valerie Hendrickson says the toxicity limits may not be achievable.

The Clean Air Task Force, an advocacy group that worked to overturn the old rule, is continuing its review of the lengthy proposed rule prior to the public comment deadline of 5 July 2011. “I’m glad it’s as strong as it is,” says senior counsel Ann Weeks. “But the devil is in the details.”

## Figures and Tables

**Figure:**